# Deep Reinforcement Learning-Based Resource Allocation for Satellite Internet of Things with Diverse QoS Guarantee

**DOI:** 10.3390/s22082979

**Published:** 2022-04-13

**Authors:** Siqi Tang, Zhisong Pan, Guyu Hu, Yang Wu, Yunbo Li

**Affiliations:** 1 Command & Control Engineering College, Army Engineering University of PLA, Nanjing 210007, China; tangsiqi3036@163.com (S.T.); huguyu@189.cn (G.H.); 18252059269@163.com (Y.L.); 2 Beijing Information and Communications Technology Research Center, Beijing 100036, China; 13218082261@163.com

**Keywords:** channel allocation, deep reinforcement learning, power control, various QoS, Satellite Internet of Things, transfer learning

## Abstract

Large-scale terminals’ various QoS requirements are key challenges confronting the resource allocation of Satellite Internet of Things (S-IoT). This paper presents a deep reinforcement learning-based online channel allocation and power control algorithm in an S-IoT uplink scenario. The intelligent agent determines the transmission channel and power simultaneously based on contextual information. Furthermore, the weighted normalized reward concerning success rate, power efficiency, and QoS requirement is adopted to balance the performance between increasing resource efficiency and meeting QoS requirements. Finally, a practical deployment mechanism based on transfer learning is proposed to promote onboard training efficiency and to reduce computation consumption of the training process. The simulation demonstrates that the proposed method can balance the success rate and power efficiency with QoS requirement guaranteed. For S-IoT’s normal operation condition, the proposed method can improve the power efficiency by 60.91% and 144.44% compared with GA and DRL_RA, while its power efficiency is only 4.55% lower than that of DRL-EERA. In addition, this method can be transferred and deployed to a space environment by merely 100 onboard training steps.

## 1. Introduction

Satellite Internet of Things (S-IoT) [1] is a wireless communication scenario where the Internet of Things (IoT) terminals transmit data through satellites. IoT devices are sparsely deployed in rural areas, including forest, mountains and the sea, with low financial efficiency. Due to the geographical limitation, it is unrealistic to deploy a Narrowband Internet of Things (NB-IoT) network in the ocean or mountain areas. Satellite constellations can be a promising plan to serve IoT devices in remote rural areas. For instance, satellite constellations such as Orbcomm and ARGOS have already played an indispensable role in the industry application of remote areas, including mining, oil and gas exploitation, forest fire prevention, disaster prediction, and environment protection.

Despite its promising prospect, S-IoT still faces technical challenges posed by not merely the limitations of satellite communication but by traits of the IoT scenario. This paper focuses on the resource allocation problem [2], whose challenges can be listed as follows.

- To satisfy the requirements on delay, reliability, the bandwidth of various IoT applications, diverse Quality of Service (QoS) guarantee [3] is a major issue for transmission mechanisms in IoT systems. More specifically, various types of services in S-IoT make it necessary for its resource allocation algorithm to pay attention to diverse QoS requirements of each data transmission.
- Compared with conventional human user terminals, the IoT terminals located in remote areas are usually battery powered and are thus energy limited, whose energy consumption of data transmission is usually inversely proportional to their lifetime. Therefore, balancing energy saving and transmission rate promotion is necessary when allocating IoT terminals’ transmission power resources.
- Compared with terrestrial IoT networks, S-IoT achieves wider coverage and supports massive IoT terminals. As the computational complexity of most resource allocation algorithms is substantially proportional to the number of users, data transmission of large-scale terminals [4] imply challenges for dynamic resource allocation.
- In a S-IoT scenario, it is always impractical to obtain perfect global Channel State Information (CSI). First, the channel quality of S-IoT, whose spectrum band is usually on Ka, is sensitive to weather conditions, especially to rainfall and snow, leading to a dynamic transmission environment of S-IoT. Due to the long satellite transmission link, the reported channel quality information tends to expire due to delay. Moreover, there are still considerable errors in the channel estimation methods despite the effort of researchers. As a result, the aforementioned errors in CSI should be considered in resource allocation.

The challenges mentioned earlier have already captured the attention of scientific communities. Although much work on resource allocation has examined how to ensure QoS requirements, deal with time-varying channel quality, save energy, and serve largescale terminals, most of the existing research has the following limitations.

- Most of the existing QoS-guaranteed resource allocation methods concentrate on a specific type of QoS metric and is implemented on homogeneous terminals in a local area. Nevertheless, considering the tens of kilometers of satellite coverage, resource allocation methods in S-IoT should cope with diverse QoS requirements of massive amounts of terminals.
- Quite a few researchers have proposed energy-saving resource allocation methods, whereas they usually focus on the energy efficiency of satellites in a downlink scenario rather than the energy-saving issue of the terminals.
- The existing model-based methods, limited by the paradigm of optimizing a specific communication model, are difficult to satisfy the intermittent access of massive terminals. The complexity of the optimization algorithm increases exponentially with the scale, resulting in difficulties in the real-time allocation of large-scale terminals. Conversely, data transmission of remote IoT terminals is usually intermittent with a limited duration, showing that the transmission resource application arrives in an online manner. However, the model-based methods need to set a fixed number of user terminals in the initialization process and are thus incapable of such online problems.
- The model-based methods usually build on specific channel model assumptions or use accurate global CSI as the input of methods. For the first set of methods, when the real-world channel is different from the predefined channel model due to interference or weather changes, their performance may severely degrade error. For the first set of methods that include channel quality, noisy and outdated CSI will lead to an accumulation of errors.

With regard to the limitations of model-based methods, an intelligent method based on deep reinforcement learning (DRL) has been introduced to the resource allocation field [5]. The essential idea of DRL is in making decisions based on the observation of the dynamic environment and adjusting the strategies according to environmental feedback, indicating that DRL-based resource allocation methods can adjust their strategies with the variation of channel quality [6]. Furthermore, the DRL, professionally designed to solve sequential decision problems, is naturally suitable for the online resource allocation problem and tends to promote the sum of long-term system rewards, rather than immediate revenue. If the method only focuses on short-term objectives, it may fall into local optima. Such long-term optimization, which is accomplished by discount factors and the updated formula of the value function, is the main advantage that makes DRL successful in sequential decision problems.

Taking advantage of DRL, we propose an online energy-saving uplink resource allocation method with diverse QoS guarantees for S-IoT. The proposed method in this paper is the first to simultaneously address diverse QoS constraints, massive terminal burst data transmission, and energy efficiency of terminals. The method adopts DRL to construct an online resource allocation pipeline for large-scale terminals. More specifically, for each emerging transmission request, the intelligent agent collects its contextual information and then simultaneously allocates channel and transmission power. The agent can promote long-term energy efficiency for S-IoT by learning from the feedback of the actual environment to guarantee diverse QoS.

The main contributions of the proposed method can be summarized as follows:

- The resource allocation problem in S-IoT was modeled to promote long-term energy efficiency with various QoS requirements. Taking the energy efficiency of remote IoT terminals into consideration prolongs the S-IoT terminals’ lifetime. Furthermore, QoS requirements include the terminals’ diverse requirements on delay, reliability, and transmission rate rather than on a certain QoS constraint. This model is more suitable with the S-IoT scenario where heterogeneous IoT applications exist in considerably broad coverage of the satellite.
- The massive terminals’ resource allocation problem is formulated as the Markov Decision Process (MDP). In response to currently generated terminal requests of each time stick, such an online resource allocation framework is more consistent with the actual practice of IoT systems where terminals always transmit data intermittently. In addition, this method’s computational complexity remains unchanged with the increase in IoT terminals, as this pipeline only concerns the currently generated requests, regardless of the other existing IoT terminals.
- A DRL-based method is proposed to solve the diverse QoS resource allocation problem. The agent observes contextual information, including channel quality, data transmission amount, terminal location, and QoS requirement to make an online channel and power allocation decisions. The agent can learn from the feedback of the environment and adjust its strategy when the channel quality or transmission traffic changes. The learning process of the agent does not depend on channel model assumptions or accurate global CSI.
- A deployment method based on a transfer learning mechanism was proposed to facilitate implementation in a large-scale LEO satellite constellation. By fixing the first several convolutional layers and fine-tuning the last layers, the converged DRL network in the simulation environment can be efficiently transferred to the actual space environment, thus reducing the computation expense and promoting system efficiency considerably.

## 2. Related Work

Large-scale resource allocation for massive IoT devices is one of the challenging issues that has attracted much attention in S-IoT [7]. Furthermore, De Sanctis et al. provided an overview discussion on QoS management and resource allocation [8].

Several works have focused on energy-saving issues in the research field of S-IoT resource allocation. Considering the limited onboard energy of LEO in the S-IoT scenario, Zhao et al. addressed the energy-saving channel allocation problem with battery load constraints by adopting DRL and taking the normalized power efficiency and service blocking rate criteria as reward function components [9]. Li et al. poured their main attention into the energy-limited remote IoT terminals in rural areas rather than on the energy-saving issue of satellites [10]. This paper adopted unmanned aerial vehicles (UAVs) as relays and then proposed an energy-efficient model by jointly optimizing channel selection, uplink transmission power control, and UAV relay deployment.

QoS is as critically vital as the energy-saving issue in S-IoT, which has been discussed from different views and approaches in recent decades. Transmission rate and delay are frequently considered QoS requirements for bandwidth and delay-sensitive services, such as environmental management. Jia et al. proposed a delay constraint joint power and bandwidth allocation algorithm by analyzing the interactions among inter-beam interference, delay factor, channel conditions, and traffic demand [11]. Furthermore, Liu et al. focused on a NOMA-based satellite industrial IoT system and proposed a power proportion optimization method for beams and nodes to guarantee the QoS, namely transmission delay and transmission rate [12]. Different from the aforementioned deterministic QoS constraints, effective capacity, which holds statistical QoS guarantee, is a more feasible alternative of QoS requirement with the consideration of the variable quality of the satellite channels. Considering the delay requirements of S-IoT devices, Yan et al. employed effective capacity to express delayed QoS requirements and developed a dynamic power allocation strategy for NOMA by the DRL algorithm [13]. Power allocation factors for NOMA users are selected dynamically by DRL in each time stick to maximize sum effective capacity while meeting each user’s minimum capacity demand constraint. Although this method solved the problems of delay and transmission rate constraints, it ignored network reliability indicators, which are of decisive importance for control applications such as position reporting in vessel navigation. Another limitation of existing studies is that they are designed to handle specific QoS requirements for applications of the same type. However, there are usually IoT devices of heterogeneous applications, and thus with diverse QoS demand, to access the same satellite in the S-IoT scenario.

As an essential factor influencing communication performance, CSI has constantly captured researchers’ attention in the resource allocation field of S-IoT. However, in most existing research, CSI is usually treated as a random variable consisting of large-scale and small-scale fading.

Therefore, it is difficult to adjust the resource allocation strategy according to the specific channel quality of a certain moment. There are three alternative ideas to solve this problem. One is allocating resource flexibly according to a specific channel model, which is adopted by Jia et al. [11] Nevertheless, they had to repeatedly build the model and optimize the problem in case the channel quality model changes [14,15], which may be caused by weather variations or external interference. The second solution, adopted by Sun et al., is leveraging a deep neural network (DNN) to approximate the SIC decoding order in NOMA-based S-IoT since the queue state and channel state continually changes [16]. Although such DNN-based methods are efficient and accurate, their accuracy depends on generating training data with the same distribution as real-world data, which is an arduous task.

Furthermore, DRL is the third method to tackle dynamic channel quality. Hu et al. first introduced DRL to the satellite resource allocation and proposed a dynamic channel allocation method for GEO satellites to decrease long-term blocking probability and improve spectrum efficiency [17,18]. Then, a multi-agent reinforcement learning-based bandwidth allocation of each beam was presented to satisfy the varying traffic demand [19]. Zhang et al. studied power allocation and drew support from DRL to adjust each beam’s transmit power according to the varying traffic demands in cache queue and channel conditions [20]. In summary, the success of the above DRL-based spectrum and power allocation methods can be attributed to the interference management and dynamic environmental perception ability of DRL. DRL-based methods [21] allocate resources according to the current specific channel quality and traffic demand, which is similar to optimization or DL-based methods. Furthermore, DRL-based methods can continuously adjust the allocation strategy intuitively to promote long-term reward according to the environment’s feedback. Such characteristics provide them with the following two advantages over DL or optimization-based ones. The first is that DRL-based methods can perform real-time adjustments with dynamic channel quality, which is changing continuously. Although the channel quality is also regarded as the observation of the environment, the DRL method uses DNN to perceive the environmental information, which shows strong robustness to noisy and error data. Therefore, it does not completely rely on the accuracy of CSI to ensure performance. The second is their long-term reward promotion of online multiple decision problems, which is precisely needed in large-scale S-IoT terminal access. However, the DL approach focuses more on current benefits, which may lead to a local optimum when solving online problems.

## 3. System Model and Optimization Formulation

### 3.1. System Model

**Figure 1:**
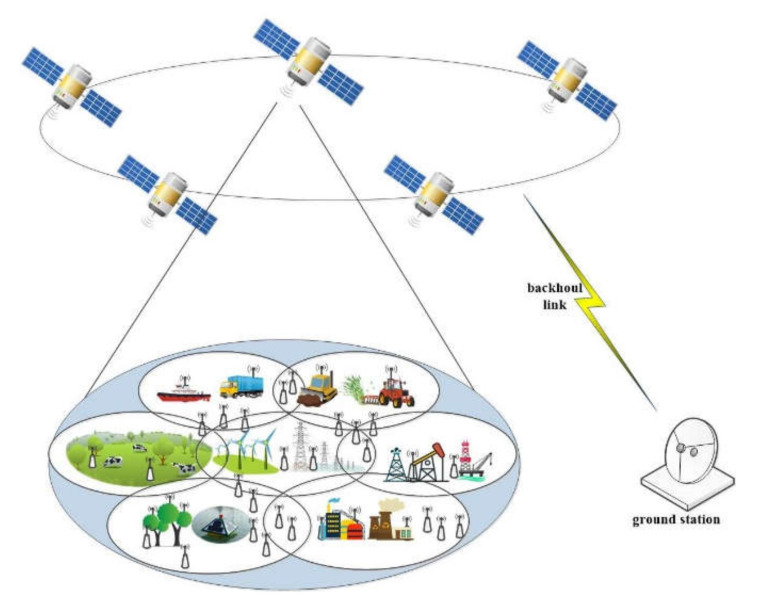
S-IoT scenario based on multibeam LEO satellite constellation.

This paper explores an uplink scenario where the multibeam LEO constellation provides services for heterogeneous remote IoT terminals with various QoS requirements. As shown in Figure 1, each multibeam of LEO satellites provides data transmission services for remote IoT terminals within its beam coverage, such as forest fire monitoring sensors and hydrological sensors. As network access selection has not been studied in this paper, it is assumed that all IoT terminals access their nearest satellite. Therefore, for IoT terminals, it can be assumed that there is only one satellite above it, regardless of other satellites in the LEO constellation. LEO satellites share the spectrum of the Ka-band and implement a direct forwarding mechanism. Furthermore, another assumption in this scenario is that the terminal remains stationary and is equipped with a single antenna.

Multiple power amplifiers on satellites receive uplink signals from IoT terminals, which are located in the geographical area of multiple corresponding beams. Let *K* and *N* denote the number of IoT terminals and beams, respectively. Consequently, the sets of IoT terminals and beams can be denoted by $U=\left\{u_{k}|k=1,2,\cdots ,K\right\}$ and $B=\left\{b_{n}|n=1,2,\cdots ,N\right\}$, respectively. More specifically, the terminal’s beam allocation depends on their location and can be represented by $x_{k}=\left[x_{k,1},x_{k,2},\cdots x_{k,N}\right],x_{k,n}\in \left\{0,1\right\}$, where $x_{k,n}=1$ denotes that terminal $u_{k}$ is located in the coverage of the nth beam and accordingly transmits data to the nth antenna element of the satellite. The maximum forwarding power of a single beam antenna is $P_{B}$, while the maximum forwarding power of the whole satellite is $P_{total}$.

The overall transmission process of $u_{k}$’s intermittent uplink data transmission request can be divided into the following processes. Firstly, $u_{k}$ employs a control channel to send access requests in the access process, containing its specific QoS requirements $QoS_{k}$ and the amount of data $D_{k}$ to be transmitted. Then, the Centralized transmission control unit (CTCU) on the satellite will take charge of channel assignment and power control to allocate specific channel $c_{k,S}$ and power $p_{k,S}$ for $u_{k}$. Subsequently, $u_{k}$ transmits its data with power $p_{k,S}$ on channel $c_{k,S}$ in the data transmission process. This paper focuses on the joint channel allocation and power control decision in the second step of the process.

For spectrum resources, the total bandwidth is divided into an independent control channel and M data transmission channels according to the frequency division multiple access (FDMA) paradigm. The available channel set for transmission can be denoted as $C=\left\{c_{m}|m=1,2,\cdots ,M\right\}$, where each channel enjoys the bandwidth of B. Therefore, the channel allocation aims to select a channel from the channel set C for IoT terminal $u_{k}$, which can be denoted as a channel allocation vector $w_{k}={\left[w_{k,1},w_{k,2},\cdots w_{k,M}\right]}^{T}$$w_{k,m}\in \left\{0,1\right\}$, where $w_{k,m}=1$ indicates that the mth channel $c_{m}$ is allocated to IoT terminal $u_{k}$. Hence the channel allocation result of all the terminals can be expressed as $W=\left[w_{1},w_{2},\cdots ,w_{M}\right],W\in {\mathbb{R}}^{K\times M}$.

In terms of power resource, $P_{k}$ denotes the maximum transmission power of the IoT terminal $u_{k}$. Though power is theoretically a continuous variable, it is usually discretized in practice in satellite communication scenarios to reduce the computational complexity of a satellite control system. We thus assume that transmission power of IoT terminals is selected from the power set of $N_{p}$ power levels, which can be denoted as $P_{set}=\left\{\frac{1}{N_{p}}P_{k},\frac{2}{N_{p}}P_{k},\cdots ,\frac{N_{p}-1}{N_{p}}P_{k},P_{k}\right\}$. According to the above definition, the uplink power control problem is also resolved in this paper. An appropriate power level has been selected for $u_{k}$ from the power set $P_{set}$. Such result of power allocation can be denoted in vector manner as $p_{k}={\left[p_{k,1},p_{k,2},\cdots p_{k,N_{P}}\right]}^{T},p_{k,n_{P}}\in \left\{0,1\right\}$, where $p_{k,n_{p}}=1$ denotes that the transmission power of IoT terminal $u_{k}$ is $p_{k,S}=\frac{n_{p}}{N_{p}}P_{k}$. The power allocation result of K terminals can then be denoted as $P=\left[p_{1},p_{2},\cdots ,p_{K}\right],P\in {\mathbb{R}}^{K\times N_{p}}$.

In summary, for IoT terminals, the channel and power allocation matrix, W and P, represent their power and channel allocation results, respectively. 

From IoT terminal $u_{k}$ to bth satellite antenna, the entire link gain can be modeled as

(1) $$G_{k,b}=G_{k}g_{k,b_{n}}(t)G_{b}\left(\varphi_{k,b_{n}}\right)$$

In Equation (1), $G_{k}$ is the transmit antenna gain of terminal $u_{k}$, while $G_{b}\left(\varphi_{k,S}\right)$ represents the receiving gain of satellite antenna, where $\varphi_{k,S}$ is the angel between terminal $u_{k}$ and the antenna center of $b_{k}$. Furthermore, $g_{k,b}\left(t\right)$ is the channel power gain and can be given by $g_{k,b}\left(t\right)=PL_{k}{\left|h_{k,b}\left(t\right)\right|}^{2}$, where $PL_{k}$ is the large-scale fading component and $h_{k,b}\left(t\right)$ captures all time-varying small-scale fading effects. The small-scale fading is composed of multipath fading and atmospheric attenuation of the satellite link, such as gaseous absorption, cloud attenuation, and rain attenuation Those time-varying components are affected by ever-changing weather and atmospheric conditions.

According to the denotations mentioned above, the received Signal to Interference plus Noise Ratio (*SINR*) [17] of terminal $u_{k}$ can be expressed as

(2) $$SINR_{k}=\frac{p_{k}G_{k,b_{k}}}{\sum_{j=1,j\neq k}^{K}w_{j,c_{k}}p_{j}G_{j,b_{k}}+\sigma^{2}}$$

Consequently, following [19], the transmission rate achieved by terminal $u_{k}$ is given as

(3) $$\begin{array}{cc} C_{k} & =B\log_{2}\left(1+SINR_{k}\right)\\ & =B\log_{2}\left(1+\frac{p_{k}G_{k,b_{k}}}{\sum_{j=1,j\neq k}^{K}w_{j,c_{k}}p_{j}G_{j,b_{k}}+\sigma^{2}}\right) \end{array}$$

### 3.2. Diverse QoS Constraints of Multiple Requests

In remote S-IoT scenarios, requests of heterogeneous devices have different QoS requirements. For stream transmission services, such as continuous data collection and surveillance services in the forest, QoS requirement is mainly on the available data transmission rates, which should exceed data emerging rates to avoid data discarding. Furthermore, monitoring services, such as disaster identification, agricultural machinery control, and industrial monitoring, may have stringent requirements on delay and reliability. As various QoS requirements on transmission rate, latency, and reliability should be simultaneously guaranteed for multiple types of services in remote S-IoT, we analyze the diverse QoS constraints in this section.

#### 3.2.1. Transmission Capacity Requirement

For real-time services such as video surveillance or continuous data collection, their QoS requirement on data transmission rate can be illustrated as

(4) $$C_{k}⩾C_{req,k}$$

where $C_{req,k}$ is the minimum transmission rate requirement of the service $s_{k}$ of terminal $u_{k}$.

#### 3.2.2. Reliability Requirement

For reliability demanding services, we employ the outage probability as the transmission reliability metric to be guaranteed. With outage threshold $\gamma_{0}$ and tolerable outage probability $p_{k}^{O}$ of service $s_{k}$, the reliability requirement can be expressed as,

(5) $$P\left\{SINR_{k}⩽\gamma_{0}\right\}⩽p_{k}^{O}$$

According to [22], reliability constraint in Equation (5) can be transformed into

(6) $$SINR_{k}⩾\gamma_{\mathrm{eff}}=\frac{\gamma_{o}}{\ln\left(\frac{1}{1-p_{k}^{O}}\right)}$$

#### 3.2.3. Latency Requirement

Due to long communication links, satellite networks should not be expected to provide ultra-low delay guarantee within 1 ms, as 5G terrestrial facilities do. However, S-IoT should still provide multiple levels of delay guarantee for various delay-sensitive services. The latency requirement of services is denoted as $T_{req,k}$ for terminal $u_{k}$’s service $s_{k}$.

Strictly speaking, different from the transmission rate and reliability requirements, the end-to-end delay of S-IoT data transmission is not only decided by a resource allocation strategy, but by the routing algorithm as well. The intuitive idea to satisfy end-to-end delay requirements is to guarantee each independent process. This paper, therefore, focuses on the latency guarantee of the uplink transmission process between the remote IoT terminal $u_{k}$ and the satellite, which mainly consists of transmission latency and signal propagation latency. The uplink latency, denoted as $T_{k}$, can be presented as:

(7) Tk=TP+LkCk

where $L_{k}$ and $C_{k}$ are the traffic data size and transmission rate of terminal $u_{k}$, respectively. In addition, $T_{p}$ is the propagation delay of signal transmitting. Decided by the distance between satellite and terminal $u_{k}$, $T_{p}$ can be approximately regarded as a constant value due to the high satellite orbit.

Thus, the latency requirement can be written as $T_{k}⩽T_{req,k}$, where $T_{req,k}$ is the maximum tolerable uplink latency of the service $s_{k}$ provided by terminal $u_{k}$.

## 4. Problem Formulation

The S-IoT uplink resource allocation algorithm needs to employ limited channel and power resources to guarantee IoT services’ diverse QoS requirements and promote system performance on the following objectives. Terminal power efficiency needs to be optimized to prolong the terminals’ lifetime. Conversely, the algorithm intuitively needs to maximize the probability of successful data transmission from IoT terminals.

The energy efficiency of terminal $u_{k}$ is defined as:

(8) $${\mathrm{EE}}_{k}^{t}=\frac{C_{k}^{t}}{p_{k,S}^{t}}$$

where $C_{k}^{t}$ represents the transmission capacity of terminal $u_{k}$ in the tth time stick, which may fluctuate according to channel quality and co-channel interference of other recently allocated terminals $p_{k}$ denotes the transmit power of terminal $u_{k}$.

For transmission success index, $A_{k}^{t}$ indicates whether the data transmission request of $u_{k}$ succeeds, which depends on whether the *SINR* exceeds the threshold.

(9) $$A_{k}^{t}=\left\{\begin{array}{lll} 0, & if & SINR_{k}^{t}<\delta_{th}\\ 1, & if & SINR_{k}^{t}\geq \delta_{th} \end{array}\right.$$

The optimization problem of the QoS-guaranteed uplink power and spectrum allocation for S-IoT terminals is formulated in Equation (10).


(10) $$\begin{array}{c} opt.\;P_{1}=\max\sum_{t=0}^{T-1}\sum_{k=1}^{K}A_{k}^{t}\;\\ \;\;\;\;\;P_{2}=\max\sum_{t=0}^{T-1}\sum_{k=1}^{K}EE_{k}^{t}\\ s.t.\;\;\;\sum_{k=1}^{K}p_{k,S}^{t}\;\leq P_{total}^{\max},\forall t\\ \;\;\;\;\;\;\;\sum_{k=1}^{K}x_{k,n}p_{k,S}^{t}\;\leq P_{B}^{\max},\forall t\forall n\\ \;\;\;\;\;\;\;C_{j}^{t}\geq \delta_{th},\forall t\forall u_{j}\in U_{On}^{t}\\ \;\;\;\;\;\;\;\sum_{n_{p}=1}^{N_{p}}p_{k,n_{p}}=1,\;p_{k,n_{p}}\in \left\{0,1\right\},\forall t,\forall k\in U\\ \;\;\;\;\;\;\;\sum_{m=1}^{M}w_{k,m}=1,\;w_{k,n}\in \left\{0,1\right\},\forall t,\forall k\in U\\ \;\;\;\;\;\;(4)(6)(7) \end{array}$$

The optimization objectives $P_{1}$ and $P_{2}$ in the above problem represent the maximization of the long-term transmission success rate and power efficiency, respectively. The first constraint indicates that the sum of the total uplink power of all terminals should not exceed the satellite’s capacity, as the satellite’s maximum forwarding power limits the sum of the uplink power received by the antenna in the direct forwarding mechanism. Similarly, the second constraint limits the uplink power of all terminals in each beam. The third constraint demands that the current resource allocation result should not interfere with the normal transmission of the existing terminals (i.e., the co-channel interference should not exceed the threshold). The following two constraints denote that only one channel and one power level are allocated to each terminal. The last three constraint items represent the heterogeneous QoS requirement on transmission rate, delay, and reliability, respectively.

The formulated problem is hard to be directly optimized for the following reasons. First and foremost, the transformation from the long-term optimization objective to a series of sub-problems on time slots needs strenuous effort. Second, the channel and power allocation indicators are binary variables, resulting in a mixed-integer nonlinear programming problem with multiple constraints. In addition, the perfectly known real-time CSI, which is the normal premise of the conventional optimization method, is unrealistic in the S-IoT scenario due to the long transmission delay. Consequently, an intelligent model-free DRL-based approach is proposed in this paper to solve the formulated problem, as further elaborated in Section 5.

## 5. DRL-Based Online Resource Allocation

The original problem in Equation (10) is first formulated through MDP in this section. Afterward, a DRL-based resource online allocation algorithm called DRL-QoS-RA is elaborated, including action space, state space, immediate reward, and operating process. We also propose a transfer learning-based deployment mechanism in Section 5.4 to reduce computational complexity and promote efficiency.

### 5.1. Algorithm Framework

To address the challenges of massive terminals’ intermittent transmission requests, this paper employs an online pipeline. The resource allocation problem is defined in Equation (10) as a sequence decision-making problem driven by data transmission requests rather than by the conventional maintenance pipeline of resource allocation methods. At each time stick, currently generated terminal requests will be sequentially allocated resources according to the dynamic environment.

Such an online resource allocation problem can be intuitively regarded as a sequential decision problem and further formulated to MDP, in which an agent in CTCU makes a decision to maximize the long-term reward according to the changing environment.

### 5.2. Mechanism of DRL

Section 5.1 illustrates the necessity and advantage of the online mechanism and further briefly introduces the MDP problem. This section will further elaborate on the essential elements of the MDP problem and DRL method, namely action space, state space, and immediate reward.

#### 5.2.1. Action Space

The intelligent resource allocation agent decides the terminal’s transmission power and channel for S-IoT terminals. More specifically, the joint resource allocation action can be intuitively defined as $a=\left\{a_{p},a_{c}\right\}$, where $a_{p}$ and $a_{c}$ denotes the power and channel allocation action, respectively. Consistent with notation in Section 3, the action space can be denoted as $C_{set}\times P_{set}$, where $C_{set}=\left\{c_{m}|m=1,2,\cdots ,M\right\}$ and $P_{set}=\left\{\frac{1}{N_{p}}P_{k},\frac{2}{N_{p}}P_{k},\cdots ,\frac{N_{p}-1}{N_{p}}P_{k},P_{k}\right\}$ represent the available channel set and transmit power set of the terminals, respectively. As a result, the size of the action space is $A=M\times N_{p}$.

Since the online allocation pipeline (as illustrated in Section 5.1) is adopted to sequentially allocate resources to the newly generated data transmission, each decision only focuses on the terminal of the current request, rather than allocating resources to each terminal. Thus, the action space is defined for the current terminal.

#### 5.2.2. State Space

In addition to the action space, another essential issue in DRL is constructing appropriate state space, which can be divided into the following two steps, choosing the related information and constructing these elements to feature the input of the DNN. Based on the analysis in Section 3, in the S-IoT scenario, the state $s_{t}$ should include the information of channel quality, current arrival terminal $u_{k,t}$, and the terminal set $U_{t}^{On}$, which contains terminals that have been allocated resource and are still transmitting data to the satellite.

The information of the current arrival request $u_{k,t}$ consists of its location $cord_{k}$, QoS requirement $Q_{k_{t}}$, and its maximum transmission power $P_{k}$, which can be denoted as ${\mathrm{info}}_{k_{t}}=\left\{cord_{k},P_{k},Q_{k_{t}}\right\}$.

For terminals that are still transmitting data to the satellite, namely $k_{t}^{On}\in U_{t}^{On}$, the involved information includes their location $cord_{k_{t}^{On}}$, QoS requirement $Q_{k_{t}^{On}}$, and the occupied channel $c_{k_{t}^{On}}$ and transmission power $p_{k_{t}^{On}}$. Therefore, for services $k_{t}^{On}\in U_{t}^{On}$, the state information is denoted as ${\mathrm{info}}_{k_{t}^{On}}=\left\{cord_{k_{}^{On}},c_{k_{t}^{On}},p_{k_{t}^{On}},Q_{k_{t}^{On}}\right\}$.

Based on the above analysis, the state at time stick t can be expressed as $s_{t}=\left\{CSI_{t},{\mathrm{info}}_{k_{t}},{\mathrm{info}}_{U_{t}^{On}}\right\}$. Note that although the agent’s observation of the environment includes CSI, there can be noise and error without the assumption that global CSI is perfectly known.

Then, to make the feature better observed by the DNN, we reformulate the elements of state information from structural representation to tensor manner, as shown in Algorithm 1.
**Algorithm 1:** Formulate State Information.Divide all the in-service terminals into M groups as the interference merely occurred between co-channel terminals, as $U_{c_{m}}^{t}$.For m=0, 1, ……M: $\mathrm{Rank}\;\mathrm{the}\;\mathrm{terminals}\;\mathrm{using}\;\mathrm{channel}\;c_{m}$$\;\mathrm{according}\;\mathrm{to}\;\mathrm{the}\;\mathrm{distance}\;\mathrm{between}\;\mathrm{them}\;\mathrm{and}\;\mathrm{the}\;\mathrm{current}\;\mathrm{arrival}\;\mathrm{request}’s\;\mathrm{terminal}\;u_{k}^{t}$$.\;\mathrm{Get}\;\mathrm{the}\;\mathrm{top}\;K_{N}$$\;\mathrm{nearnest}\;\mathrm{terminals}\;\mathrm{as}\;U_{c_{m}}^{t}$. $\mathrm{If}\;U_{c_{m}}^{t}\neq \emptyset$: $\;\mathrm{For}\;\mathrm{each}\;\mathrm{terminal}\;u_{j}\in U_{c_{m}}^{t}$:   Construct $u_{j}$$’s\;\mathrm{contextual}\;\mathrm{information}\;D_{u_{j}-u_{k}^{t}},p_{j,S}^{t-1},D_{k},Csi_{j,S}^{t-1},C_{j}^{t-1},EE_{j}^{t-1},Rc_{j}^{t},$$Rd_{j}^{t},Rr_{j}^{t}$$\;\mathrm{as}\;\mathrm{feature}\;\mathrm{vector}\;s_{j}^{t}$$,\;\mathrm{where}\;Dis_{u_{j}-u_{k}^{t}}$$\;\mathrm{denotes}\;\mathrm{the}\;\mathrm{distance}\;\mathrm{between}\;u_{k}^{}$$\;\mathrm{and}\;u_{j}$$,\;p_{j,S}$$\mathrm{denotes}\;\mathrm{the}\;\mathrm{transmission}\;\mathrm{power}\;\mathrm{of}\;u_{j}$$,\;D_{k}$$\;\mathrm{denotes}\;\mathrm{the}\;\mathrm{data}\;\mathrm{transmission}\;\mathrm{amount}\;\mathrm{of}\;u_{j}$$,\;Csi_{j,S}^{t-1}$$\;\mathrm{denotes}\;\mathrm{the}\;\mathrm{data}\;\mathrm{transmission}\;\mathrm{of}\;\mathrm{last}\;\mathrm{time}\;\mathrm{stick},\;EE_{j}^{t-1}$$\;\mathrm{denotes}\;\mathrm{the}\;\mathrm{energy}\;\mathrm{efficiency}\;\mathrm{of}\;\mathrm{last}\;\mathrm{time}\;\mathrm{stick},\;Rc_{j}^{t}$$,\;Rd_{j}^{t}$$,\;\mathrm{and}\;Rr_{j}^{t}$$\;\mathrm{denotes}\;\mathrm{the}\;\mathrm{QoS}\;\mathrm{requirement}\;\mathrm{on}\;\mathrm{transmission}\;\mathrm{rate},\;\mathrm{delay}\;\mathrm{and}\;\mathrm{reliability}\;\mathrm{of}\;\mathrm{terminal}\;u_{j}$ respectively.  $\mathrm{Arrange}\;\mathrm{the}\;\mathrm{state}\;\mathrm{information}\;\mathrm{vector}\;s_{j}^{t}$$\;\mathrm{of}\;\mathrm{the}\;K_{N}$$\;\mathrm{terminals}\;\mathrm{as}\;a\;\mathrm{matrix}\;s_{c_{m}}^{t}\in {\mathbb{R}}^{K_{N}\times 9}$$,\;\mathrm{which}\;\mathrm{contains}\;\mathrm{the}\;\mathrm{contextual}\;\mathrm{information}\;\mathrm{on}\;\mathrm{channel}\;c_{m}$ at time stick t. $\mathrm{Construct}\;\mathrm{the}\;\mathrm{state}\;\mathrm{information}\;\mathrm{matrix}\;s_{c_{m}}^{t}$$\;\mathrm{of}\;\mathrm{each}\;\mathrm{channel}\;\mathrm{to}\;\mathrm{tensor}\;\mathrm{manner}\;\mathrm{and}\;\mathrm{get}s_{ts}^{t}\in {\mathbb{R}}^{K_{N}\times 9\times M}$ as the input of DQN 

Through the above process, the state information $s_{t}=\left\{CSI_{t},{\mathrm{info}}_{k_{t}},{\mathrm{info}}_{U_{t}^{On}}\right\}$ is formulated as a $K_{N}\times 9\times M$ tensor.

#### 5.2.3. Immediate Reward

To maximize the terminals’ long-term energy efficiency with diverse QoS requirements guaranteed, we attach the QoS constraints to the objective of Equation (10) and reconstruct the objective as

$$O=\sum_{t=0}^{T-1}\left(a_{1}\sum_{k=1}^{K}EE_{k}^{t}+\sum_{k\in U_{1}}G\left(a_{2}\left(C_{k}^{t}-C_{req,k}\right)\right)+\sum_{k\in U_{2}}G\left(a_{3}\left(SINR_{k}^{t}-\gamma_{\mathrm{eff}}\right)\right)+\sum_{k\in U_{2}}G\left(a_{4}\left(T_{k}^{t}-T_{req,k}\right)\right)\right)$$

G(x) is the piece-wise function, whose expression is,

(11) $$G(x)=\left\{\begin{array}{l} A,x⩾0\\ x,x<0 \end{array}\right.$$

The objective is composed of four parts. The first one corresponds to the terminals’ energy efficiency, while the second to the fourth one indicate the penalty of unsatisfied QoS requirement on transmission rate, outage probability, and latency. The purpose of weight $a_{1}$ is to balance the promotion of revenue and the penalty of QoS unsatisfaction, whereas $a_{2}\cdots a_{4}$ aim to normalize the penalty parts. This long-term objective can be divided into that of each time stick in the process of online resource allocation, which can be expressed as

(12) $$O_{t}=a_{1}\sum_{k=1}^{K}EE_{k}^{t}+\sum_{k\in U_{1}}G\left(a_{2}\left(C_{k}^{t}-C_{req,k}\right)\right)+\;\sum_{k\in U_{2}}G\left(a_{3}\left(SINR_{k}^{t}-\gamma_{\mathrm{eff}}\right)\right)+\sum_{k\in U_{2}}G\left(a_{4}\left(T_{k}^{t}-T_{req,k}\right)\right)\;$$

To present short-term benefits achieved by making a resource allocation decision for a specific arrival terminal service, we intuitively adopt $\Delta O_{t}=O_{t+1}-O_{t}$ as the immediate reward to inform the agent how much the total goal increases or decreases because of action $a_{t}$.

Notice that if the action can’t satisfy the QoS requirements of arrival service and existing services, the action will not be carried out, meaning that the resource will not be allocated. The reward of the actions is thus equivalent to

(13) $$r_{t}=\left\{\begin{array}{l} a_{1}\left(\sum_{k=1}^{K}EE_{k}^{t+1}\;\;-\;\sum_{k=1}^{K}EE_{k}^{t}\;\right)\;\;\;\;\;\;\;\;\;\;\;\;\;\;\;\;\;\;\;\;\;\;\;\;\;\;\;\;\;\;\;\;\;\;\;\;\;\;\;\;\;\;\;\;\;if\;a_{t}\in \Phi_{satisfy}^{t}\\ \sum_{k\in U_{1}}G\left(a_{2}\left(C_{k}^{t}-C_{req,k}\right)+a_{3}\left(SINR_{k}^{t}-\gamma_{\mathrm{eff}}\right)+a_{4}\left(T_{k}^{t}-T_{req,k}\right)\right)\;\;if\;a_{t}\notin \Phi_{satisfy}^{t} \end{array}\right.$$

where $\Phi_{satisfy}^{t}$ denotes the action set which can satisfy the QoS requirement of existing and arrival services at time stick t.

### 5.3. Process of DRL-Based Online Resource Allocation

**Figure 2:**
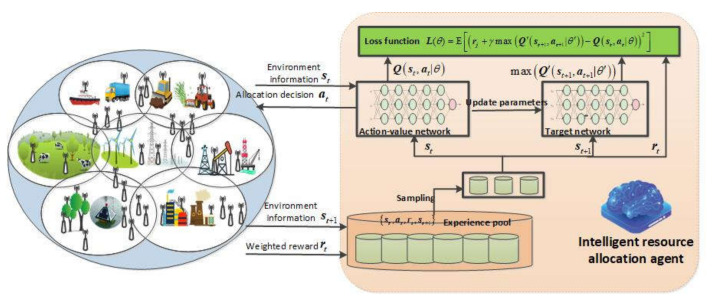
Framework of DRL_CAPC algorithm.

Following the conventional training pipeline of DRL, this section presents the training process of the proposed DRL-QoS-RA algorithm. As illustrated in Figure 2, the agent observes environmental information $s_{t}$, including channel information, QoS requirement, data amount, and existing terminals’ resource occupation. Then based on a certain policy π, the resource allocation action $a_{t}$ is determined, and then the current reward $r_{t}$ is collected, with the environment changing to $s_{t+1}$. Each time the agent goes through the above process, the experience $\left\{s_{t},a_{t},r_{t},s_{t+1}\right\}$ is collected and put into the experience pool, from which the agent can periodically sample experience data and train the policy neural network to optimize long-term benefits. The converged strategy neural network promotes energy efficiency and transmission success rate with QoS requirements guaranteed.

The goal of this paper is to optimize long-term reward.

(14) $$\underset{\pi }{\max}\sum_{t=0}^{T}r_{t}$$

However, since the system operates continuously rather than in a limited time, RL’s classical theory adds the discount factor γ to Equation (14) to make the long-term reward more meaningful. $\gamma \in \left(0,\left.1\right]\right.$ is used to adjust the short-term and long-term impact [23], in other words, how far the agents consider when making decisions. In terms of intuitive perspective, it makes the agent pay more attention to the impact of its action on the near future state. From the perspective of the agent’s training process, $T_{len}=1/(1-\gamma )$ can be used to estimate the number of steps that the agent considers in the future when making decisions. For t>1/(1−γ), the discount parameter $\gamma^{t}$ is almost 0. It means that the agent’s action selection does not consider its influence on the state beyond the T range, which benefits the convergence of RL methods. Therefore, with discount factor γ, the optimization goal is

$$V^{\pi }\left(s\right)=E\left[\underset{t_{0}}{\sum^{t_{0}+T_{len}}}\;\gamma^{t-t_{0}}r_{t}\right]$$,

which indicates that the agent optimizes the long-time revenue by optimizing the discounted reward of the future T steps at each time stick. As a result, the larger that γ is, the more steps the agent takes into consideration with more difficulty of the training process. The smaller that γ is, the more the agent pays attention to the immediate interests. The long-term revenue to be optimized can be denoted as:

(15) $$\max_{\pi }\;E\left[\underset{t=0}{\sum^{T}}\;\gamma^{t}r_{t}\right]$$

where $E\left[\cdot \right]$ denotes expectation and π denotes a specific strategy.

For a certain state, the optimization objective is defined as the state value function in RL theory $V^{\pi }\left(s\right)$, which denotes the long-term reward of a specific strategy from state s. Owing to Markov property, $V^{\pi }\left(s\right)$ can be represented as

(16) $$V^{\pi }\left(s_{t}\right)=r_{t}+\gamma \sum_{s_{t+1}}P\left(s_{t+1}\left|s_{t},\pi \left(s_{t}\right)\right.\right)V\left(s_{t+1},\pi \right)$$

In RL theory, the state-action value of action $a_{t}$ and state s is also defined as $Q\left(s_{t},a_{t}\right)$, which is the accumulated reward if action $a_{t}$ is chosen by the agent.

(17) $$Q^{\pi }\left(s_{t},a_{t}\right)=r_{t}+\gamma \sum_{s^{\prime }\in S}\;P\left(s_{t+1}\left|s_{t},a\right.\right)V^{\pi }\left(s_{t+1}\right)$$

The goal of RL agent is to learn to perform actions to maximize the sum of benefits received in the long term. To achieve this goal, RL methods use different ways to estimate the state value function $V^{\pi }\left(s\right)$ or state-action value function $Q^{\pi }\left(s,a\right)$. For example, Q learning, as a popular method of RL, adopts a Q value table to estimate $Q^{\pi }\left(s,a\right)$.

When the state space becomes huge, the value function approximation methods based on table form encounter a dimensional disaster problem and are thus no longer applicable. To solve this problem, a deep neural network is implemented as the mapping function from state to value $V^{\pi }\left(s\right)$ or $Q^{\pi }\left(s,a\right)$. RL methods that approximate value function through a deep neural network are called ‘deep RL’ (DRL), which are still based on the classical theory of RL, but facilitate deep learning to replace the original tabular value function estimation module.

Deep Q-network (DQN) [24] is a popular method of DRL, which improves the Q-learning method by introducing a neural network to estimate the state action value $Q^{\pi }\left(s,a\right)$ instead of a Q -value table. The deep neural network in DQN is called the Q network, whose parameter of neurons is denoted by *θ*. The input of the Q network is the state *s_t_*, while the output of the network is the Q value of each action under the state *s_t_*. More specifically, the Q value of each state–action pair $\langle s_{t},a_{t}\rangle$ denotes the long-term reward of choosing the action $a_{t}$ in state $s_{t}$.

The essential idea of DQN is the same with Q learning, which is to determine the Q value of each action under a specific state when the state transition probability *P* is unknown, so as to obtain the optimal decision. The optimal strategy is to choose the action with the largest Q value.

(18) $$\pi^{*}(s_{t})=\underset{a\in A}{\arg\max}Q_{}^{*}\left(s_{t},a\right)$$

With this strategy, the state value of $s_{t}$ can be denoted as

(19) $$V^{\pi }\left(s_{t}\right)=\max_{a\in A}Q_{}^{*}\left(s_{t},a\right)$$

The optimal Q value can be obtained by iteratively calculating Equations (17) and (19). More specifically, the estimated value of *Q* network can be calculated by

(20) $$y_{t}=r_{t}+\gamma \underset{a_{t+1}\in A}{\max}\hat{Q}\left(s_{t+1},a_{t+1}\left|\theta^{-}\right.\right)$$

Therefore, the difference between the estimated and prediction value of *Q* network can be denoted as

(21) $$L(\theta )=E\left[{\left(y_{t}-Q\left(s_{t},a_{t}|\theta \right)\right)}^{2}\right]$$

This section adopts a gradient decent method to train the Q network, with *L*(*θ*) employed as a loss function. The procedures of the DRL-based online resource allocation algorithm (DRL-QoS-RA) are elaborated in Algorithm 2.

Therefore, by learning the Q network, the network can select the action that can maximize the V value according to the current state, which is $\max_{\pi }E\left[\sum_{t=0}^{T}\;\gamma^{t}r_{t}\right]$. The optimization of the long-term reward can be realized by making decisions in this way at every moment. The agent pays attention not only to the immediate rewards concerning the current allocation to the current terminal, but also to the long-term impact of its occupied resources.
**Algorithm 2:** Implementation of DRL-QoS-RA.Parameter initialization. Initialize network Q$\;\mathrm{and}\;Q^{\prime }$ with random weight. 
$\mathrm{Initialize}\;\mathrm{DRL}\;\mathrm{training}\;\mathrm{parameters},\;\mathrm{such}\;\mathrm{as}\;\mathrm{target}\;\mathrm{network}\;\mathrm{update}\;\mathrm{step}\;N_{Q}$, greedy exploration probability ε$,\;\mathrm{Buffer}\;\mathrm{size}\;N_{B}$$,\;\mathrm{replay}\;\mathrm{start}\;\mathrm{size}\;N_{IS}$$,\;\mathrm{and}\;\mathrm{batch}\;\mathrm{size}\;N_{BS}$. Initialize the S-IoT environment parameter. For time_stick t=1,T do$\;\mathrm{Collect}\;\mathrm{new}\;\mathrm{data}\;\mathrm{transmission}\;\mathrm{request}\;\mathrm{into}\;\mathrm{set}\;U_{new}^{t}$$\;\mathrm{Collect}\;\mathrm{the}\;\mathrm{suspended}\;\mathrm{data}\;\mathrm{transmission}\;\mathrm{into}\;\mathrm{set}\;U_{On}^{t}$ and release the resource occupied by them.$\;\mathrm{If}\;U_{new}^{t}\neq \emptyset$:$\;\;\mathrm{For}\;\mathrm{each}\;\mathrm{data}\;\mathrm{transmission}\;\mathrm{request}\;u_{k}^{t}$,$\;\;\mathrm{Reformulate}\;\mathrm{state}\;\mathrm{information}\;s_{t}=\left\{CSI_{t},{\mathrm{info}}_{k_{t}},{\mathrm{info}}_{U_{t}^{On}}\right\}$$\;\mathrm{into}\;\mathrm{tensor}\;\mathrm{manner}Feature_{k,t}$.$\;\;\mathrm{Calculate}\;a\;\mathrm{candidate}\;\mathrm{action}\;\mathrm{set}\;\Phi_{satisfy}^{t}$$\;\mathrm{Adopt}\;\mathrm{the}\;\mathrm{tensor}\;\mathrm{feature}\;Feature_{k,t}$ as the input of the action−value network Q and obtain the output of network Q$\;\mathrm{as}\;v_{a_{t}}=Q\left(s_{ts}^{t}|\theta \right)$ With probability 1−ε$\;\mathrm{to}\;\mathrm{decide}\;a_{t}=\arg\max v_{a_{t}}$$,\;\mathrm{otherwise}\;\mathrm{randomly}\;\mathrm{choose}\;a_{t}$.$\mathrm{If}\;a\in \Phi_{satisfy}^{t}$$\;:\;\mathrm{allocate}\;\mathrm{resource}\;\mathrm{for}\;u_{k}^{t}$$\;\mathrm{according}\;\mathrm{to}\;a_{t}$.$\mathrm{Collect}\;\mathrm{environmental}\;\mathrm{information}\;s_{t+1}$$,\;\mathrm{then}\;\mathrm{formulate}\;\mathrm{to}\;Feature_{k,t+1}$$\;\mathrm{and}\;\mathrm{calculate}\;r_{t}$$\mathrm{Store}\;\mathrm{this}\;\mathrm{experience}\;\left(Feature_{k,t},a_{t},r_{t},Feature_{k,t+1}\right)$ into experience pool DIf the sample of D$\;\mathrm{is}\;\mathrm{more}\;\mathrm{than}\;\mathrm{this},\;\mathrm{replay}\;\mathrm{start}\;\mathrm{size}\;N_{IS}$:$\;\mathrm{Sample}\;N_{BS}$$\;\mathrm{experience}\;\left(Feature_{k,t},a_{t},r_{t},Feature_{k,t+1}\right)$ from D$\;\mathrm{Calculate}\;y_{t}$$\;\mathrm{and}\;L\left(\theta \right)$ by Equation (20) and Equation (21) respectively. Update the weights of Q$\;\mathrm{by}\;\mathrm{minimizing}\;L\left(\theta \right)$$\mathrm{Every}\;N_{Q}$$\;\mathrm{steps},\;\mathrm{update}\;\hat{Q}$ using weight of Q

### 5.4. Deployment Mechanism Based on Transfer Learning

Despite its advantages in learning from the feedback of the environment, the proposed DRL-QoS-RA method still has limitations when deployed in the S-IoT system. The reason can be attributed to the time and calculation expense of the DRL method’s training process, which is further illustrated as follows:

- If the training process is implemented on the satellite, and the system suffers from the agents’ decision-making errors during the training process, which will result in the failure of IoT data transmission and a waste of system resources. If the training process is implemented in the simulation environment of the ground control center, the accuracy of the algorithm will suffer from the difference between the simulation environment and the actual onboard environment.
- Each satellite of a large-scale LEO constellation faces a unique environment to learn from. Thus, the computational expense of training can be tremendous if added up in a massive satellite constellation.

Concerning the shortcomings mentioned above, this section embraces transfer learning and proposes a deployment mechanism for DRL-QoS-RA, whose essential idea is that the filters of deep CONV network are learned to observe the environment feature and can be adapted (or transferred) to another similar environment.

As present in Algorithm 3, by fixing the first several CONV layers, the knowledge learned by the agent in the simulation environment can be preserved. Then, by fine-tuning the last fully connected layers, the Q network can adapt to the target environment. Compared with direct deployment, merely fine-tuning the fully connected layers can reduce the computation expense and promote system efficiency.

**Algorithm 3:** Deployment process of DRL-QoS-RA.Build simulation environment in ground control center based on the historical channel state, terminal location and IoT request distribution.$\mathrm{Train}\;\mathrm{the}\;\mathrm{model}\;Q\left(Feature_{k,t}\left|\theta \right.\right)$ to convergence in a simulation environment For satellite = 0, 1, 2……: 

Copy the weight θ


 of the DQN Q

 

$\mathrm{Fix}\;\mathrm{the}\;\mathrm{conv}\;\mathrm{layers}\;\mathrm{in}\;\mathrm{the}\;\mathrm{deep}\;\mathrm{convolutional}\;\mathrm{network}\;\mathrm{and}\;\mathrm{only}\;\mathrm{fine}-\mathrm{tune}\;\mathrm{the}\;\mathrm{last}\;\mathrm{few}\;\mathrm{convolutional}\;\mathrm{layers}\;\mathrm{until}\;\mathrm{the}\;\mathrm{agent}\;\mathrm{converges}\;\mathrm{to}\;\mathrm{obtain}\;Q\left(Feature_{k,t}\left|\theta^{\prime }\right.\right)$



## 6. Simulation Result and Analysis

In this section, the S-IoT environment and DRL parameters are introduced first. Then, the results and analysis of transmission success rate and energy efficiency are given. Finally, the performance of the transfer learning-based deployment mechanism is illustrated.

### 6.1. Experiments Establishment

#### 6.1.1. Scenario Parameters

In this paper, a multibeam LEO satellite is used to simulate the experimental environment of S-IoT, whose detailed parameters are listed in Table 1. The limited backhaul transmission power of the satellite limits the IoT terminals that the system can support.

Furthermore, in the simulation environment, 10,000 IoT terminals are unevenly distributed in the total beams of a single satellite, whose request arrival follows the Poisson distribution with *λ* times per hour.

#### 6.1.2. DRL Training Parameters

The adopted DQN structure is a convolutional neural network with an input layer, two convolutional layers and two fully connected layers. The conv layers consist of 16 3 × 3 filters and 32 2 × 2 filters. The number of neurons in the hidden layer is 128 and 48, while the ReLu is utilized as the activation function. All other parameters related to the DQN are listed in Table 2. Note that the listed parameters are selected from multiple simulation tests to balance the complexity and performance of the DRL algorithm.

#### 6.1.3. Comparative Methods

To evaluate the performance of the proposed DRL-QoS-RA algorithm, this section compares it with the following methods,

(1) Genetic algorithm: In the GA-based [25,26] online resource allocation method, the normalized weighted objective. $\alpha_{1}P_{1}+\alpha_{2}P_{2}$ is adopted as the optimization objective with the number of parents *N_p_* = 200, probability of variation *p_M_* = 0.005, crossover probability *p_C_* = 0.05, and number of iterations *N_I_* = 800.
(2) DRL-EERA: DRL-based Energy-efficient resource allocation method. DRL-EERA improves the method proposed in [3] by taking power control into consideration, as [3] only considers the channel allocation problem. More specifically, DRL_EERA adopts the state representation and instant reward in [3], while adding power control in action space to allocate transmission power and channel simultaneously. The training parameters of DRL are consistent with the DRL-QoS-RA method, where the deep neural network includes four layers, namely two convolution layers and two fully connected layers.
(3) DRL-RA: DRL-based resource allocation method. Similar to DRL-EERA, the action design was modified to simultaneously allocate power and channel based on [9]. Moreover, the training parameters and network structure are consistent with DRL-QoS-RA.
(4) Random method: Power and channel of the current terminal are randomly allocated.

### 6.2. Convergence Analysis of DRL-QoS-RA Method

**Figure 3:**
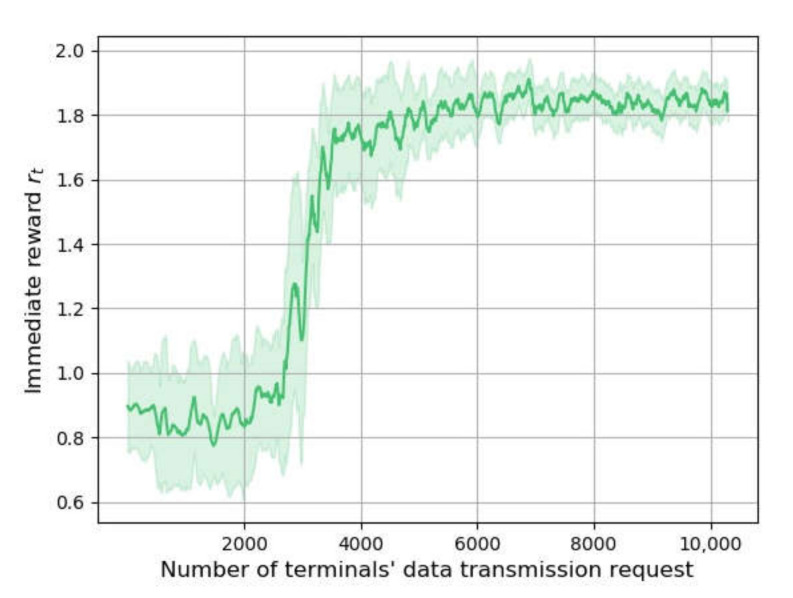
Convergence process of DRL-QoS-RA method.

Figure 3 shows the changing of reward through a training process when the terminals’ data transmission frequency is five per hour, representing the convergence effect of the methods. The horizontal axis represents the number of transmission requests, while the vertical axis indicates the value of the reward. The shaded area is drawn according to the standard deviation of *r_t_* to show its fluctuation.

The replay start size of DRL-QoS-RA is set to 2000. The agent uses random decision mechanisms for the environment exploration and experience gathering when the number of requests is below 2000. As a result, the reward is not significantly improved in this stage. Then, the value of the reward shows significant growth, indicating the improved performance of the algorithm. After the number of requests reaches 8000, the fluctuation of *r_t_* is reduced, implying the convergence of DRL-QoS-RA.

**Figure 4:**
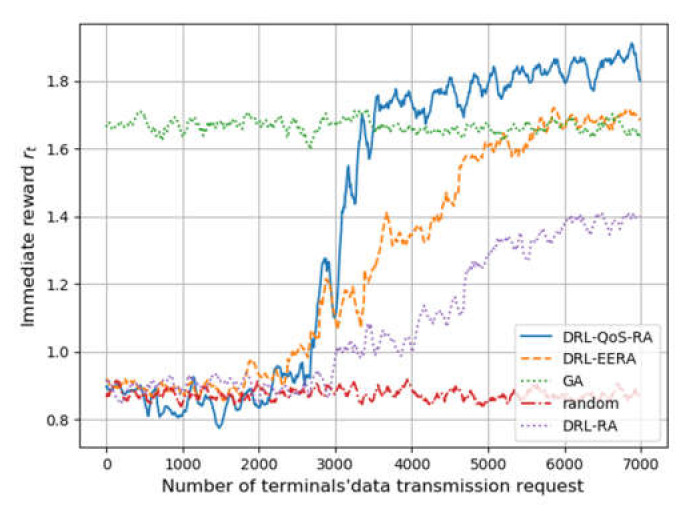
The simulation process of each methods’ training process.

Figure 4 shows each methods’ performance on the reward *r_t_*. Similar to Figure 3, the horizontal axis represents the number of transmission requests, while the vertical axis denotes reward *r_t_*, which is drawn by averaging steps of 100.

First of all, the performance of the three methods based on DRL, namely DRL-QoS-RA, DRL-RA, and DRL-EERA, is similar to that of the random mechanism before the training process of DRL. The reward of the three methods increases with the number of iterative steps.

On the training process, when the request number is between 2000 and 6000, DRL-RA and DRL-EERA take about 4500 training times to achieve convergence, while DRL-QoS-RA only needs 2000 training times. Such an improvement on convergence efficiency can be mainly attributed to the following two points: (1) Compared with DRL-EERA and DRL_RA, DRL-QoS-RA formulates the condensed feature tensor rather than the intuitive location-based zero-padding mechanism, which is used by DRL-EERA and DRL_RA. As a result, the deep network can perceive the critical information more easily and reduce the number of parameters that the deep network needs to train. (2) The state space of DRL-QoS-RA contains more contextual information, such as the amount of data to be transmitted, channel quality, and QoS requirement.

When the request number is more than 6000, all four methods obtain convergence. Compared with the GA method, the reward after convergence of DRL-RA is lower by 14.3%, and the DRL-EERA method achieves similar performance, while the reward of the proposed DRL-QoS-RA method is 11% higher than that of GA.

### 6.3. Deployment Process Simulation Based Transfer Learning

To demonstrate the adaptability of our deployment mechanism based on transfer learning, we evaluate our method in the transfer learning setting to test the transfer mechanism proposed in Section 5.4. Specifically, the network environment is changed after the 10,000th terminal request, including the quantity and distribution of terminals, their data transmission parameters, and channel quality.

**Figure 5:**
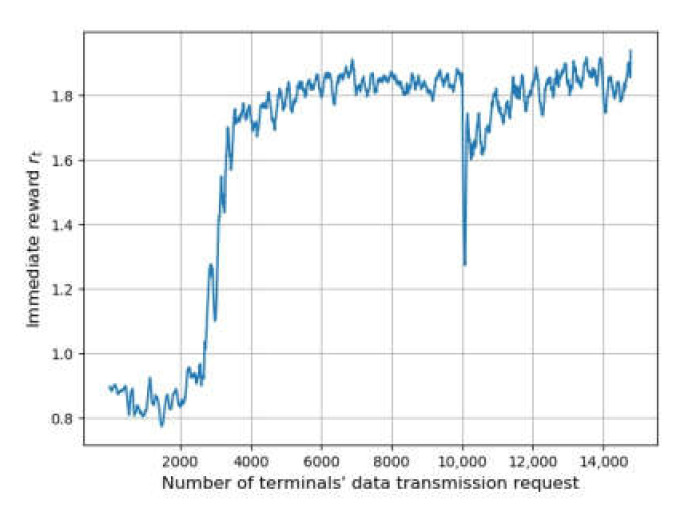
Deployment training process based on transfer learning.

As shown in Figure 5, it takes 4000 times to train DRL-QoS-RA from the initial state to converge. As for the transfer stage, only 1000 times or even about 100 times of training are needed for adapting to the new environment with different distribution and to reach acceptable performance. Furthermore, Figure 5 shows the performance of a transferred neural network. Compared with DRL-QoS-RA trained from the initial state, the transferred one achieves an approximate effect on transmission success rate and power efficiency. To sum up, the transfer mechanism can effectively reduce the onboard training time and computing expense in system deployment.

### 6.4. Transmission Success Rate and Power Efficiency

**Figure 6:**
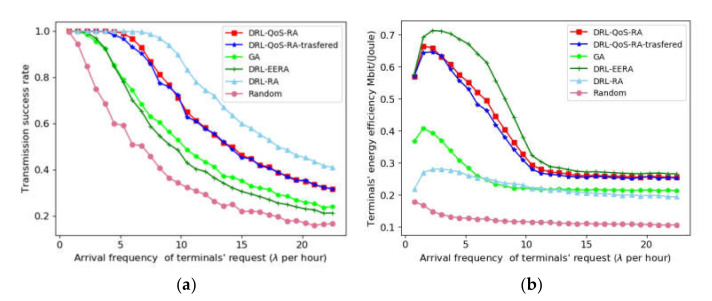
Methods’ performance with requested traffic increase. (**a**) The trend of transmission success rate with traffic increase. (**b**) The trend of energy efficiency with traffic increase.

The simulation result of transmission success rate is illustrated in Figure 6a, where the horizontal axis denotes the terminals’ average arrival and thus shows the IoT transmission traffic. When the traffic load exceeds a certain limit, the transmission success rate of all methods begins to decrease with the increase in traffic. The DRL-RA, which takes the success of the current transmission as the immediate benefit, achieves the best effect. The proposed DRL-QoS-RA algorithm can also achieve a higher transmission success rate than the DRL-EERA and GA. For example, when the transmission success rate is 0.8, DRL-RA achieves the traffic arrival rate *λ* = 10.90, whereas DRL-QoS-RA, DRL-EERA and GA can carry the traffic with the arrival rate *λ* = 8.48, *λ* = 4.87, and *λ* = 5.21, respectively. In other words, the proposed DRL-QoS-RA method can improve transmission success rate by 74.12% and 62.76% compared with DRL-EERA and GA, respectively. The reason why DRL-RA achieves better performance than the other methods may lie in the fact that its reward concentrates on success rate.

Figure 6b shows the trend of the terminals’ energy efficiency with the gain of beam traffic. With the increase in the average transmission rate of the terminal, the power utilization rates of DRL-QoS-RA, DRL-EERA, DRL-RA, and GA show a similar trend. After a slight increase, they decrease and become stable eventually. The reason for the subsequent decline stage is that with the increase in requests, co-channel interference inevitably increases, and thus the increase in throughput is less than the power consumption. Then, with the continuous increase in traffic flow, the system becomes saturated by rejecting many new transmission requests. As a result, the power utilization rate of the system appears to be stable. Concerning the power efficiency of S-IoT’s normal operation condition, when we can find that the proposed DRL-QoS-RA is 0.66 Mbit/(Joule), while that of DRL-EERA, GA and DRL-RA is 0.69 Mbit/(Joule), 0.41 Mbit/(Joule), and 0.27 Mbit/(Joule), respectively. The proposed method can improve the power efficiency by 60.91% and 144.44% compared with GA and DRL_RA, while its power efficiency is only 4.55% lower than that of DRL-EERA.

Although DRL-EERA and DRL-RA achieve the best performance in energy efficiency and success rate, respectively, the proposed DRL-QoS-RA better achieves a trade-off between energy saving and transmission QoS satisfaction.

Table 3 shows the success rate, energy efficiency and computational time of the above six optimization algorithms under different transmission frequencies. The calculation time of the GA method increases greatly with the increase in terminal request’s frequency; thus, it is not suitable for S-IoT. The calculation time of the four DRL-based methods remains stable with the increase in in-service terminal. As DRL-QoS-RA transferred adopts the transfer learning mechanism, its training steps are lower than the other three DRL-based methods and can achieve a favorable transmission success rate and energy efficiency with lower calculation cost. Similar to the performance in Figure 6, the proposed DRL-QoS-RA and DRL-QoS-RA-transferred can effectively achieve the tradeoff between energy utilization and transmission success rate. Such a promotion is presented with either low terminal transmission frequency (when S-IoT is relatively idle) or high terminal transmission frequency (when S-IoT tends to be saturated).

## 7. Conclusions

Aiming to solve the uplink channel allocation and power control problem of large-scale terminals with various QoS requirements in the S-Iot system, this paper proposes the DRL-QoS-RA method for online joint resource allocation based on DRL. Compared with conventional DRL methods, the success of DRL-QoS-RA can be attributed to (1) the comprehensive reward concerning QoS requirement, transmission success rate, and energy efficiency; and (2) contextual information, including location, resource occupation, CSI, and QoS requirement. Furthermore, a deployment mechanism based on transfer learning is proposed to facilitate practical usage in the real satellite system, effectively promoting efficiency and thus saving precious onboard computational resources.

## Figures and Tables

**Table 1:** Detailed parameters of S-IoT scenario.

Parameters	Value
Satellite altitude	550 km
Beams	19
Data transmission channels	8
Channel bandwidth	10 KHz
Frequency band	14 GHz
Terminals’ antenna max power	300 mW
Terminals’ antenna power level	3
Terminals’ antenna gain	10 dBi
Satellite receiving antenna G/T	3.7 db
Path loss	170.38 db
Satellite backhaul power limitation	300 W
Beam power limitation	25 W
Satellite amplifier magnification	5
*SINR* threshold	1.1

**Table 2:** DRL-QoS-RA algorithm parameters.

Algorithm Parameters	Value
Replay start size	2000
Replay memory	20,000
Batchsize	32
Target network update step	50
Discount factor	0.99
Initial exploration rate	1.0
Final exploration rate	0.01
Exploration rate decay	5 × 10^−4^
Learning rate	0.001

**Table 3:** Performance of comparative methods with different request arrival rates.

Methods	*λ* = 1.5			*λ* = 9.75		
	Success Rate	Energy Efficiency	Computational Time (s)	Success Rate	Energy Efficiency	Computational Time (s)
random	0.94	0.16	-	0.34	0.12	-
GA	0.99	0.41	3.24 × 10^3^	0.53	0.22	1.67 × 10^4^
DRL-RA	1	0.27	64.77	0.89	0.23	64.77
DRL-EERA	1	0.69	71.52	0.48	0.38	71.52
DRL-QoS-RA	1	0.66	78.31	0.71	0.33	78.31
DRL-QoS-RA-transferred	1	0.64	11.53	0.72	0.31	11.53

## Data Availability

No applicable.
